# The Influence of the Interlayer Distance on the Performance of Thermally Reduced Graphene Oxide Supercapacitors

**DOI:** 10.3390/ma11020263

**Published:** 2018-02-08

**Authors:** Jun-Hong Lin

**Affiliations:** Department of Mold and Die Engineering, National Kaohsiung University of Applied Sciences, Kaohsiung 80778, Taiwan; jhlin@kuas.edu.tw

**Keywords:** ionic liquids, supercapacitors, reduced graphene oxide, cationic surfactant, interlayer distance

## Abstract

In this paper, cationic surfactant cetyltrimethylammonium bromide (CTAB) was employed to prevent the restack of the thermally reduce graphene oxide (TRG) sheets. A facile approach was demonstrated to effectively enlarge the interlayer distance of the TRG sheets through the ionic interaction between the intercalated CTAB and ionic liquids (ILs). The morphology of the composites and the interaction between the intercalated ionic species were systematically characterized by SEM, SAXS, XRD, TGA, and FTIR. In addition, the performance of the EDLC cells based on these TRG composites was evaluated. It was found that due to the increased interlayer distance (0.41 nm to 2.51 nm) that enlarges the accessible surface area for the IL electrolyte, the energy density of the cell can be significantly improved (23.1 Wh/kg to 62.5 Wh/kg).

## 1. Introduction

Owing to global warming and the depletion of fossil fuels, the demand on alternative energy storage and conversion devices is growing higher than ever. As an energy storage device, the supercapacitor has drawn much attention because it has the advantages of long cycle life, high power capability, wide range of operating temperature and being maintenance free [1,2]. Supercapacitors are known to store energy in electrical double layer capacitors (EDLCs) based on ion adsorption on the electrode/electrolyte interfaces. Following the energy density Equation (1),

E = 1/2 CV^2^ (1)

Most works related to the performance of EDLC cells are focused on employing electrode materials with high specific surface area, appropriate electrolyte with wide electrochemical window, and tuning the electrode/electrolyte interface properties [1,2]. Various kinds of carbon-based conductive materials or compounds have been reported for EDLC electrodes. Among these materials, graphene is one of the promising electrode materials toward high-performance EDLC because of its unique 2-dimensional structure with high intrinsic electron mobility and large theoretical specific area (2630 m^2^/g) [3]. There are several approaches to producing graphene such as chemical vapor deposition [4,5], electric arc discharge [6], epitaxial growth on the specific surface [7] and chemical oxidation-reduction methods [8,9], etc. However, challenges remain in the preparing and processing of this 2-dimensional material. Among these approaches, the chemical oxidation-reduction method has the potential for mass production since graphene oxide (GO) can be easily obtained by the oxidation of natural graphite [9,10]. During the oxidation process, the defects and oxygen groups are introduced to the basal plane of the graphene. Thus, GO has a unique amphiphile with a negatively charged hydrophilic group and a hydrophobic basal plane. Due to the attached oxygen groups, GO is an electrical insulator and can be dispersed in water [3].

However, for EDLC applications, the removal of these oxygen groups is required to convert the GO to the highly conductive reduced graphene oxide (RGO) by a post reduction process. The hydrophobic nature of the graphene would lead to the quick aggregation of the RGO sheets; the RGO sheets would thus lose their attraction as individual objects and further processing of the composite materials would be hindered [3]. The use of surfactants is common to keep the suspension stabilized in solution while preventing the restack of the RGO sheets in solid [11,12,13]. Ionic surfactants are amphiphilic compounds made up of ionic hydrophilic head groups and extended apolar, organic residues and hydrophobic tails. Thus, surfactants could interact with RGO through the residual charged group. In addition, the hydrophobic interactions between the aliphatic chains and basal plane play an important role in the stabilization of RGO sheets in water [11]. 

On the other hand, ILs are favorable for high-performance EDLC cells not only because of their high electrochemical window but also due to their advantages of low volatility and high thermal stability. Zhang et al. [12] employed a series of ionic surfactants to stabilize the GO sheets during the reduction process. They found that the surfactants can be successfully intercalated in both GO and RGO to prevent the restack phenomena. In addition, their results indicate that for the same surfactant intercalated electrodes, the aqueous electrolyte has a larger capacitance than the IL electrolyte. This might be due to the fact that aqueous electrolytes usually have smaller comprising ions than the IL electrolytes. Thus, the aqueous solutions may take advantage of their small ion size that facilitates the ion transport in small pores. In contrast, the average ion diameter D of ILs, for example, for 1-ethyl-3-methylimidazolium bis(trifluoromethylsulfonyl)imide (EMI-TFSI) is D ~ 0.7 nm [14], which is usually larger than the reported interlayer distance of the surfactant intercalated RGO (~0.4 nm). Thus, the small interlayer distance might reduce the accessible surface area for ILs in the RGO sheets. Here, we employed thermally reduced graphene oxide (TRG) as the conductive material because it is chemical free and has few layers of graphene structure, high specific surface area, and high electrical conductivity [15,16]. IL, EMI-TFSI is chosen because of its high conductivity and low viscosity [14].

**Figure 1:**
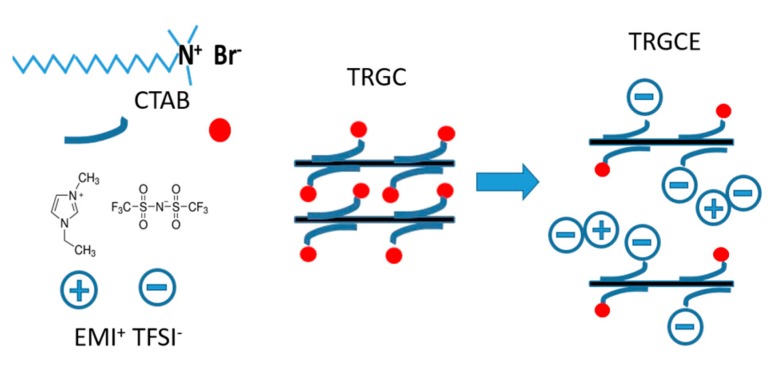
The schematic depicts the EMI-TFSI IL incorporates with the intercalated CTAB surfactant to enlarge the interlayer distance of the graphene sheets.

As shown in Figure 1, the schematic depicts the proposed process for tuning the interlayer distance between graphene sheets. The cationic surfactant CTAB was applied to intercalate in the TRG sheets forming the TRGC composite [13] and afterward the EMI-TFSI was added to interact with the TRGC during the filtration to obtain the TRGCE composite. Since both (EMI^+^) (TFSI^−^) and ionic surfactant CTAB are ionic materials, the Coulombic force between the intercalated CTAB and the EMI-TFSI might result in the large ionic aggregates or micelles in between TRGCE sheets and hence increase the interlayer distance. 

X-ray diffraction (XRD), small angle X-ray scattering (SAXS), Fourier-transform infrared spectroscopy (FTIR), and thermal gravimetric analysis (TGA) were employed to characterize the interlayer distance, the bond vibration and the compositions of these electrode materials. In addition, a VersaSTAT 4 potentiostat was employed to characterize the electrical performance of these two-electrode EDLC cells. It was found that the vibration mode of the CTAB head group was shifted due to the interaction with EMI-TFSI, implying the Coulombic or ion exchange interactions between the CTAB and EMI-TFSI [17]. Furthermore, the X-ray results revealed an interlayer distance of 0.41 nm as a result of CTAB intercalation in between the TRGC sheets. In addition, the interlayer distance of the TRGCE sheets was further enlarged to 2.51 nm suggesting the formation of CTAB/EMI-TFSI aggregates or micelles. The substantial increase in both capacitance and energy density of the TRGCE cell may mainly be attributed to the larger interlayer distance that leads to more accessible surface area for the IL electrolytes.

## 2. Experimental Procedures

**Figure 2:**
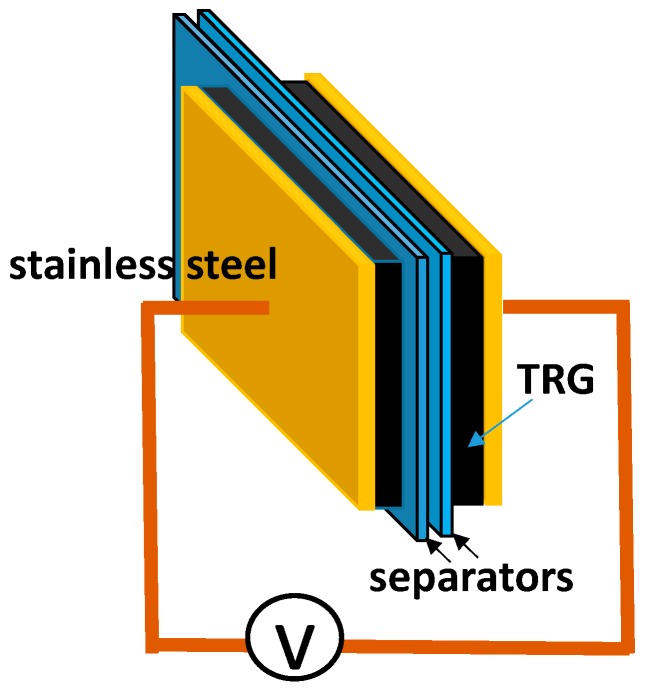
The schematic of the EDLC cell.

The thermally reduced graphene oxide (TRG) was purchased from GIBusiness company, Taiwan and was synthesized from natural graphite by a modified Hummers method [8] followed by a thermal treatment at elevated temperature. CTAB intercalated TRG termed as TRGC was prepared in the presence of CTAB surfactant in water. Normally, TRGC electrodes were obtained by dispersing 10 mg of TRG powder in 30 mL of 0.1 M CTAB solution with the aid of ultra-sonication and vigorous stirring for 12 h. After that, the TRGC solution was deposited and washed with D.I. water on a Celgard 3500 separator (Celgard LLC, Charlotte, NC, USA) by a vacuum filter. Further, the TRGC electrodes were rinsed with 15 mL of 0.2 M of EMI-TFSI ethanol solution during filtration to obtain the TRGCE electrodes. For comparison, the TRG electrodes were also prepared by dispersing 10 mg of TRG in 20 mL of 20 wt % ethanol solution with the same dispersion and filtration process. The deposited electrode was flipped over on a 1 cm^2^ of 304 stainless steel current collector for EDLC cell assembly. The EDLC cell was in the form of two-electrode package in a sealed testing bag filled with EMI-TFSI as electrolytes as shown in Figure 2. The electrical measurement was carried out with a potentiostat (VersaSTAT 4, Princeton Applied Research, Oak Ridge, TN, USA), with the uncertainty of resistance (±0.1 ohm/cm^2^). The uncertainty of the averaged capacitance and the averaged energy density are <±5%, and the error bar is presented as approximately the size of the symbol in the plots. The microstructures of the composites were characterized by scanning electron microscope (SEM; JSM-6700, JEOL, Tokyo, Japan), X-ray diffraction (XRD; Bruker AXS D8, Bruker AXS GmbH, Karlsruhe, Germany, CuKα = 1.5406 Å, with a resolution of 0.05 degrees per step) and small angle X-ray (SAXS; NanoStar, Bruker AXS GmbH, Karlsruhe, Germany, CuKα = 1.5406 Å, with a resolution of q = 0.00071 Å^−1^). The weight composition was determined by the thermal gravimetric analysis (TGA; TA Q50, TA Instrument, New Castle, DE, USA, with the weighing precision ±0.01%, at a heating rate of 10 °C/min under nitrogen flow). The bond vibration of the composites was also investigated by the Fourier transform infrared spectrometer (FTIR, Vertex 70v, Bruker Optik GmbH, Ettlingen, Germany, with a spectral resolution of 0.4 cm^−1^ in a wave number range of 4000 to 400 cm^−1^).

## 3. Discussion

**Figure 3:**
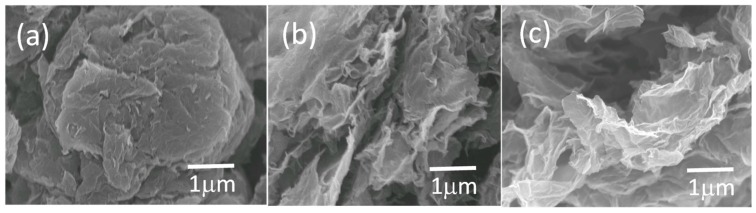
The SEM image of (**a**) TRG (**b**) TRGC and (**c**) TRGCE.

The morphology of the TRG, TRGC, and TRGCE were characterized by the SEM. As shown in Figure 3, without the intercalated CTAB, the TRG would tend to agglomerate into graphite-like big particles. In contrast, the CTAB intercalated TRGC and TRGCE have a more curved flaky central structure with a more disorderly and irregular layered structure at the wrinkled edge parts, indicating the intercalation of the CTAB.

**Figure 4:**
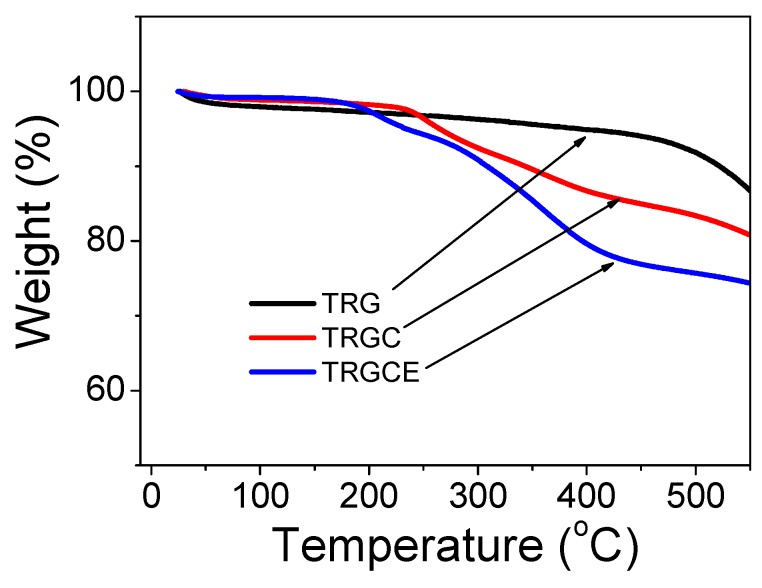
The thermal gravimetric analysis for TRG, TRGC and TRGCE composites.

The composition of neat TRG and CTAB intercalated composites TRGC and TRGCE were analyzed by the thermal gravimetric analysis (TGA). As can be seen in Figure 4, all the materials show slight mass loss around 50 °C due to the containing of moisture. The TRG exhibits a smooth weight loss from 50 °C to 500 °C with an 8% total loss at 500 °C followed by a steep weight loss due to the quick decomposition of the TRG. The TRGC shows steep weight loss from 240 °C to 400 °C due to the decomposition of the intercalated CTAB while the TRGCE starts to show significant weight loss from 190 °C to 400 °C because of the decomposition of the containing CTAB and EMI-TFSI. The total weight losses of 17% and 25% at 500 °C are observed for the TRGC and TRGCE composites, respectively. Compared to the TRGC, the increase of the weight loss of TRGCE might be ascribed to the additional EMI-TFSI that interacted with the intercalated CTAB. 

**Figure 5:**
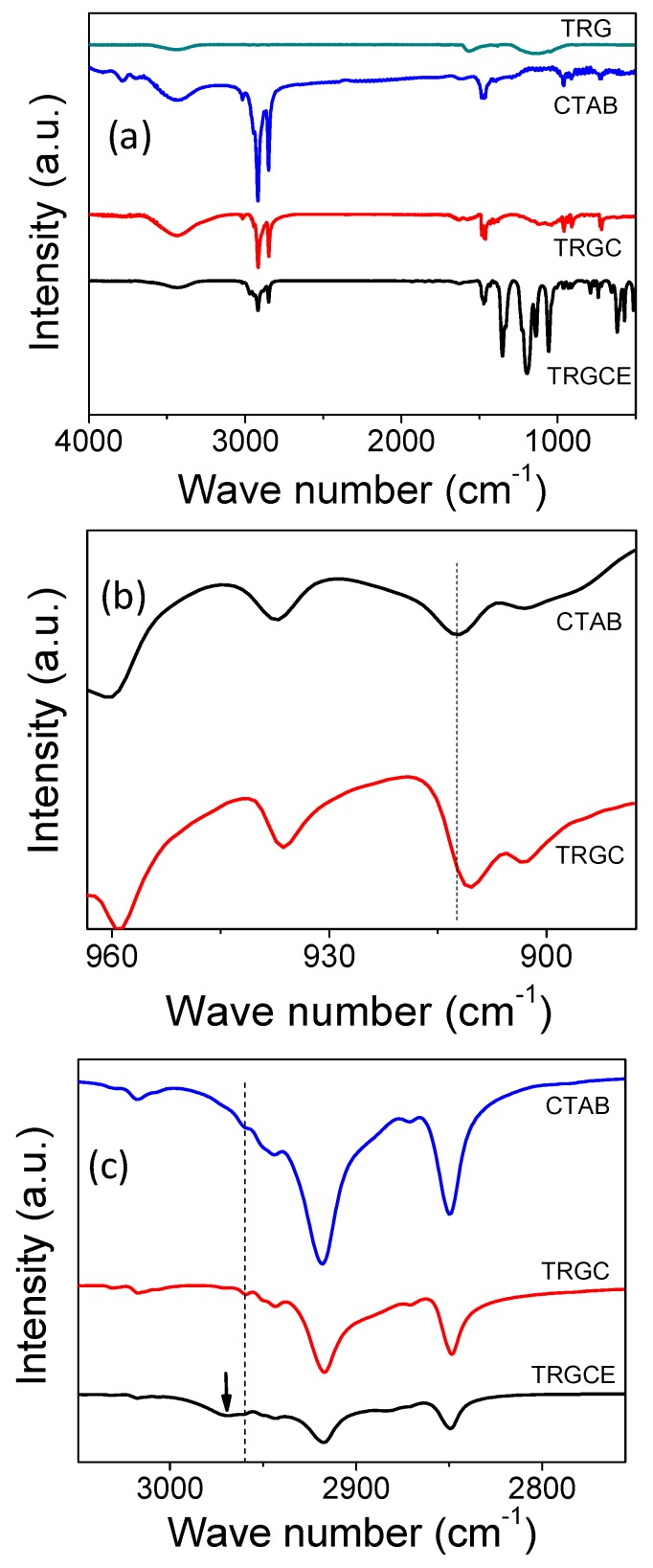
(**a**) FTIR spectra of the TRG, CTAB, TRGC, and TRGCE samples; (**b**) zoom in for CTAB and TRGC, (**c**) zoom in for CTAB, TRGC and TRGCE.

Figure 5 plots the Fourier Transform Infrared Spectrometer (FTIR) results in the wavenumber region of (4000–400 cm^−1^). For the GO sample, several vibration modes representing its specific molecular features were often reported including the bands at 3430, 1716, 1635, 1154, and 1033 cm^−1^ related to the vibration mode of the O–H, C=O, C=C, C–OH, and C–O groups, respectively [18,19]. As can be seen in Figure 5a, for the TRG, most of the oxygen-related groups are substantially removed, which was confirmed by limited signals at 1154 cm^−1^ and 3430 cm^−1^, characteristics for C–OH and O–H groups, respectively. In addition, the vibration modes corresponding to the CTAB molecules are presented providing the bases for further comparison with the TRGC and TRGCE. For the neat CTAB, the bands at 2917 cm^−1^ and 2849 cm^−1^ represent the stretching of the C–H bond and the bands at 1463 cm^−1^, and 1473 cm^−1^ are related to the scissoring of the CH_2_ group [19,20]. After CTAB intercalated into TRG substrate, in the TRGC sample, the bands at 1463 cm^−1^ and 1473 cm^−1^ related to the C–H vibration of CTAB are not significantly affected by the TRG substrate. However, the band at 912 cm^−1^ (in Figure 5b), which represents the C–N vibration of CTAB, is found slightly shifted to 910 cm^−1^ [19,20] implying the interaction between CTAB and TRG. On the other hand, for the TRGCE sample (in Figure 5c), the band at 2959 cm^−1^ [19,20] representing the vibration of the CTAB head group (N–CH_3_) is found shifted to 2969 cm^−1^. The substantial shift in the ionic head group of the intercalated CTAB might imply the ion exchange or the Coulombic interaction between the CTAB and EMI-TFSI.

**Figure 6:**
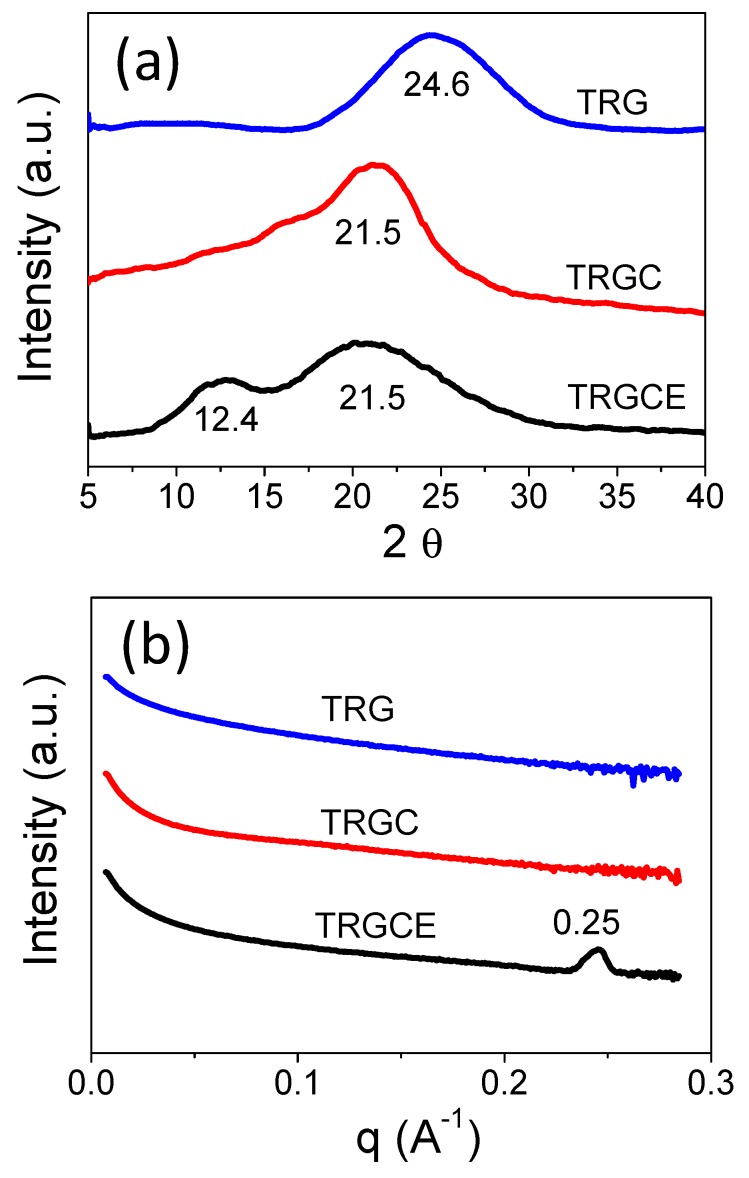
(**a**) The X-ray diffraction scattering and (**b**) small angle X-ray scattering of the TRG, TRGC and TRGCE composites.

The feature sizes of TRG, TRGC, and TRGCE are determined by both the XRD and SAXS. As can be seen in Figure 6a, without surfactant, the TRG shows (001) reflection peak at 24.6 degrees corresponding to the average interlayer distance of 0.36 nm. The interlayer distance of the intercalated sheets depends on the size of the intercalated species and the interaction forces [12]. The TRGC exhibits (001) reflection peak at 21.5 degrees. Thus, the interlayer distance of the TRGC is 0.41 nm, which is consistent with the reported result [13]. However, the increase in the interlayer distance (0.05 nm) is too small compared to the molecular size of the CTAB. It is known that the residual charged functional groups are mainly at the edge of the TRG sheet [13]. Thus, the intercalated CTAB might interact with the charged functional groups at the edge of the TRG sheets leading to the small increase in the interlayer distance.

On the other hand, compared with TRGC, the reflection intensity (at 21.5 degrees) of the TRGCE is found significantly reduced. In addition, an additional small peak is observed at a lower angle of 12.4 degrees (0.71 nm). The observed reflection peak at a lower angle represents the increased interlayer distance of the TRGCE. To confirm the existence of a larger interlayer distance that might be beyond the scope of our XRD, the SAXS measurement was also carried out. Figure 6b presents the SAXS patterns of TRG, TRGC and TRGCE samples and is expressed as intensity I vs. scattering vector q. The SAXS probes the repeating microfeatures in the distance range between few nm to few tens of nm in polymers or their composites. The repeating structure feature (d) of different shape and size can thus be estimated by the scattering vector q in Equation (2) [21],

(2) $$q=\frac{4\pi \sin\theta }{\lambda }$$

where λ is the wavelength of the X-ray. Therefore (d) can be deduced by Equation (3) [21],

(3) $$d=\frac{2\pi }{q}$$

As can be seen in Figure 6b, for the TRGCE sample at the low q region, the increase in power law decay exponent indicates the scattering of a larger size of objects [21]. 

For the TRG and TRGC samples, no specific peak is observed over the whole measured region implying that their repeating feature sizes are beyond the scope of the SAXS measurement. However, the TRGCE sample exhibits a sharp scattering peak at q = 0.25 Å^−1^, indicating an interlayer distance of 2.51 nm. Compared with the TRGC sample, the observed intensity reduction at 21.5 degrees and the additional peaks at 12.4 degrees and q = 0.25 Å^−1^ for the TRGCE sample suggests that part of the CTAB intercalated sheets interacted with the additional EMI-TFSI in the form of large-sized CTAB/EMI-TFSI complexes or micelles that enlarge the interlayer distance between TRGCE sheets.

**Figure 7:**
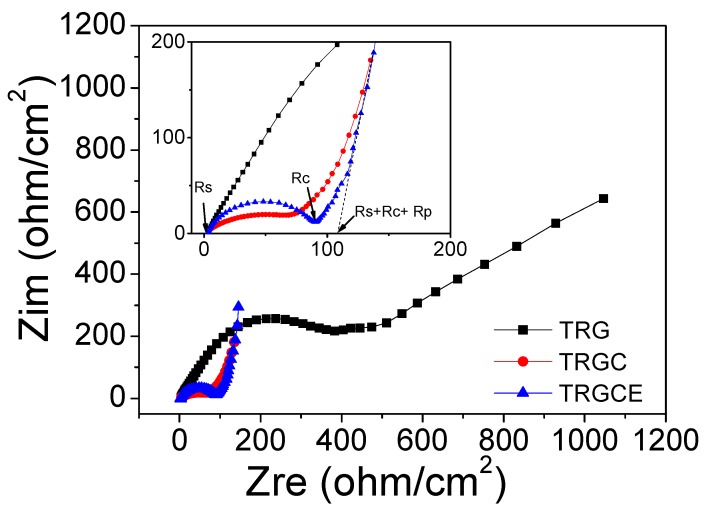
The impedance spectra of the TRG, TRGC and TRGCE EDLC cells.

Figure 7 expresses the Nyquist plot for the TRG, TRGC and TRGCE EDLC cells. For the TRGCE sample, from high to low frequency, the impedance curve shows a semi-circle followed by a transition zone transferring to a vertical line. As can be seen, the semicircle intercepts or approaches the real axis at Rs and Rs + Rc. Rs is usually attributed to the resistance of ion transport in the electrolyte. Rc is a combined resistance result of both electrode materials and electrolyte [22,23]. For the high conductive electrolyte here, the Rc is mainly attributed to the contact resistance between graphene particles and that between the graphene electrodes and the current collectors in the electrolyte. The high to medium frequency region represents the charge transfer resistance associated with the porous structure of the electrodes. As can be seen in the TRGCE curve, because of the ion diffusion mechanism between Warburg diffusion and ideal capacitive ion diffusion, the deviation from the vertical line is observed showing an inclined angle between 45 and 90 degrees against the real axis [22,24]. This nonideal capacitance response can be ascribed to the pore size distribution inducing different penetration depth of the TRGCE electrode [24]. The resistance Rp represent the Warburg related diffusion process which can be estimated by extrapolating the low-frequency data to the real axis. The x-axis intercept is equal to the internal resistant R = Rs + Rc + Rp [25,26]. As shown in the Figure 7 inset, all the samples have similar Rs value of 2.8 ohm/cm^2^. The TRG exhibits the highest Rc of 388.2 ohm/cm^2^. This might be due to the visible precipitation of TRG during the electrode preparation. The large precipitated particles could reduce the contact interface between the large particles leading to the high resistance of the TRG cells. On the other hand, at the low-frequency region, the TRG cell exhibits a clear inclined straight line. This might be due to its small interlayer distance (0.36 nm) that limited the ion diffusion of the EMI-TFSI electrolyte. The TRGCE cell (91.6 ohm/cm^2^) exhibits a higher resistance than the TRGC cell (67.6 ohm/cm^2^). This is expected because the increased interlayer distance of the TRGCE sheets might result in the loose electrical contacts between graphene sheets. In addition, the Rp of TRGCE (16.2 ohm/cm^2^) is smaller than that of TRGC (22.3 ohm/cm^2^), suggesting that the larger interlayer distance of the TRGCE sheets might facilitate the diffusion of EMI-TFSI electrolyte. The internal resistance R values of TRGC and TRGCE are therefore 92.7 ohm/cm^2^ and 110.6 ohm/cm^2^, respectively. 

**Figure 8:**
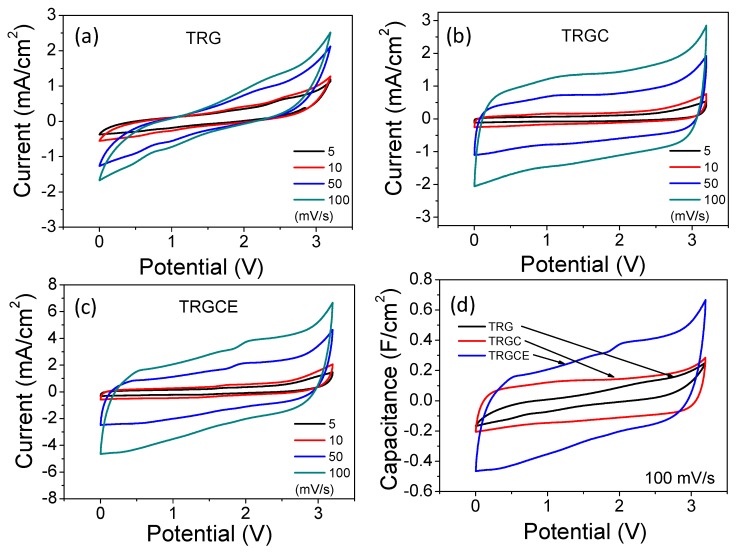
Cyclic voltammetry (CV) curves of (**a**) TRG, (**b**) TRGC and (**c**) TRGCE cells at the various voltage scan rates. (**d**) the capacitance response of the TRG, TRGC and TRGCE cells.

Figure 8 plots the cyclic voltammetry (CV) curves of the TRG, TRGC and TRGCE cells. The cell current is in response to the applied voltage (from 0 to 3.2 V) at various voltage scan rates. As can be seen, at low scan rate except for the TRG cell, both TRGC and TRGCE cells have an approximately rectangular shape showing the near-ideal capacitance behaviors. The accumulated charge Q with the applied voltage V follows the Q = CV, where C is the capacitance. Therefore, the response current I = dQ/dt = C * dV/dt, (if C is a constant) under a constant voltage scan rate, a constant current should be obtained resulting in a rectangular shape of the CV curve. However, consider that the real capacitor is usually in series with an equivalent resistor which is related to internal resistor R [27]. Thus, the response current increases or decreases exponentially with a time constant RC to a steady state current. With the increase of RC, it takes more time to reach the steady state and hence collapses the rectangular profile. In addition, increasing the scan rate means reducing the response time and that would also cause similar results to those observed in Figure 8a–c [28,29]. Based on the shape of the CV curve, the TRG should have the highest R among these samples which is consistent with the previous EIS conclusion. Figure 8d plots the capacitance against voltage profiles in which the CV current at 100 mV/s is divided by the same scan rate. As can be seen, except the TRG cell, both TRGC and TRGCE cells show a more rectangle capacitance response, indicating that they are closer to ideal capacitors. In addition, the capacitance response of these cells shows a clear sequence of TRGCE > TRGC > TRG. 

**Figure 9:**
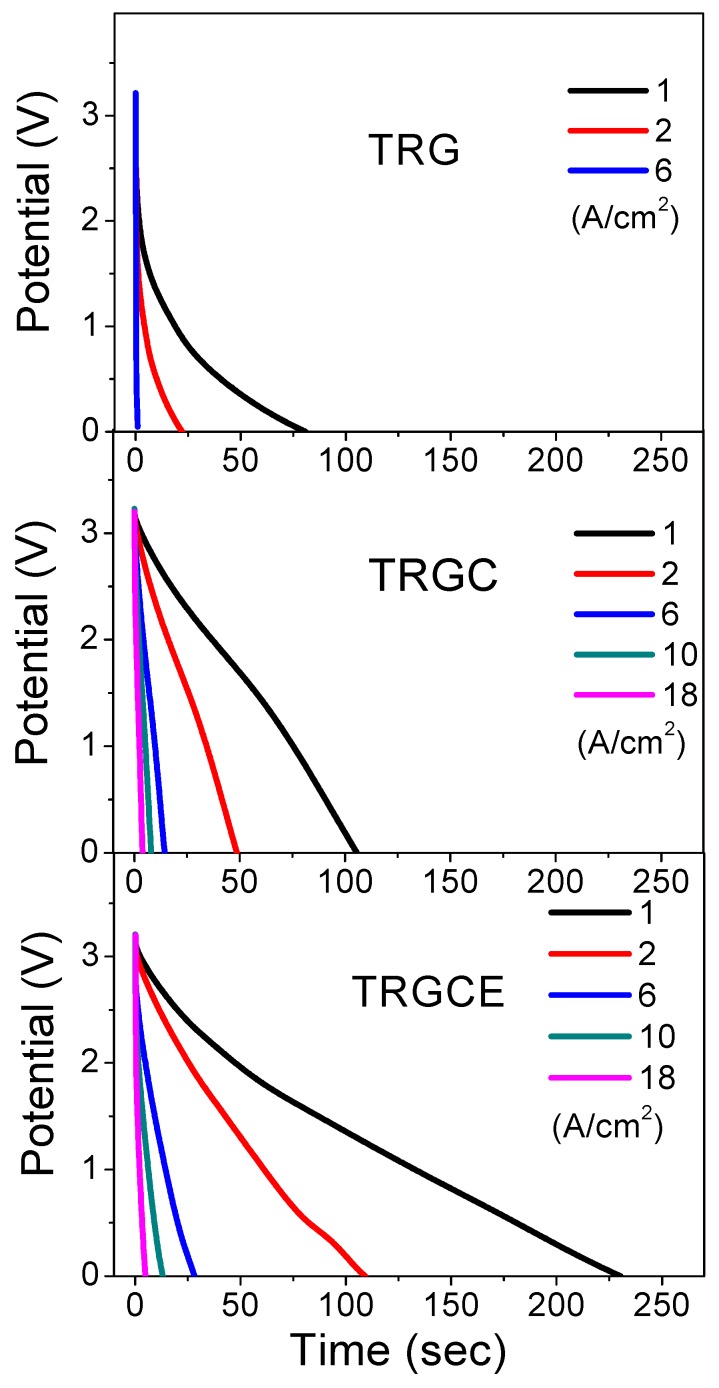
The discharge current vs. time for the TRG, TRGC and TRGCE cells at various discharging current densities.

**Figure 10:**
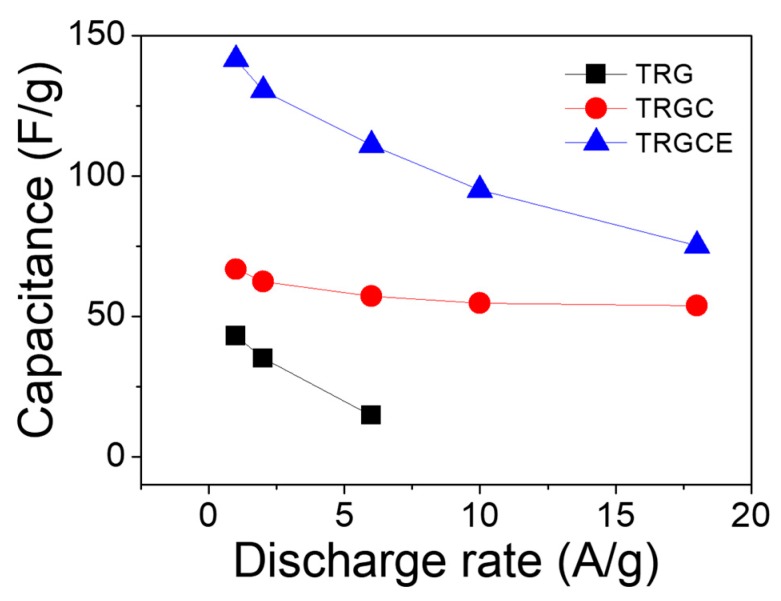
The capacitance density vs. current density of the TRG, TRGC and TRGCE cells.

Taking advantage of the high electrochemical window of EMI-TFSI, the high operation voltage (3.2 V) brings the high energy density of these cells. The galvanostatic charge/discharge for these two-electrode cells is performed at various current densities. The discharge currents of TRG, TRGC, and TRGCE cells are shown in Figure 9. As can be seen, a voltage drop at the beginning of the discharging process is assigned to the voltage loss when the current flow across the equivalent resistance. The specific capacitance Cs of the graphene composites can be extracted from the galvanostatic discharge curve following Equation (4) [30,31,32],

(4) $$C_{S}=\frac{4C}{m}=\frac{4I\Delta t}{m\Delta V}$$

where m is the total weight of the active materials on both electrodes, C is the capacitance of the cell, I is the constant current, Δt is the discharging time, and ΔV is the potential change (excluding the initial voltage drop) during the discharging. As shown in Figure 10, the capacitance response of the cell decreases as a function of the discharging current density. The decrease in capacitance at high current density is significant especially for the TRG cell. This is because the measured high current density is a result of massive ions accumulated on the electrode in a short period. Therefore, the ions may not have sufficient time to diffuse in and out of the deep region of the pores and would tend to accumulate on the surface of the electrodes, leading to the decease of accessible surface area and hence capacitance [31]. At the low current density region, the ions might have sufficient time for the accessible surface area. Therefore, the value of the specific capacitance implies the accessible surface area at the specific current density. At the low current density of 1 A/g, the specific capacitances of the TRG, TRGC, and TRGCE are 43, 67 and 141 F/g, respectively. The TRGCE has a capacitance which is 2.1 folds higher than that of TRGC, suggesting the increase of accessible surface area for the TRGCE electrodes. As expected, at the high current density region, the capacitances of the TRG, TRGC, and TRGCE dropped to 15 F/g (at 6 A/g), 54 F/g (at 18 A/g) and 75 F/g (at 18 A/g), respectively. Still, the TRGCE has the highest capacitance because of its large interlayer distance that facilitates the ion transport.

The energy densities are obtained by the following Equation (5) [30,31,32],

E = 1/2 CV^2^ = 1/8 Cs V^2^ (5)

Moreover, the power density is deduced according to Equation (6) [31,32],

(6) $$P=\frac{E}{\Delta t}$$

**Figure 11:**
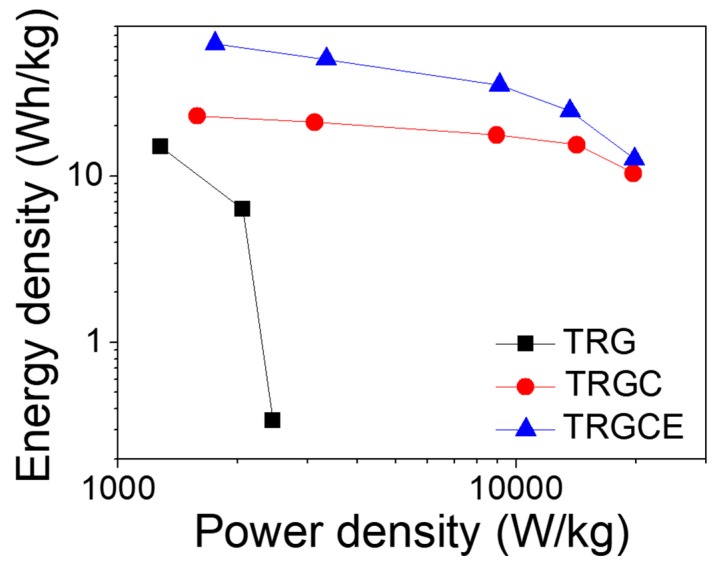
The Ragone plot of the TRG, TRGC and TRGCE cells.

Ragone plot reveals the energy density of the TRG, TRGC and TRGCE cells as a function of power density as shown in Figure 11. As can be seen (Figure 9), the large voltage drop and the small capacitance of the TRG cell result in its small energy density of 15.3 Wh/kg at 1 A/g. In contrast, both TRGC and TRGCE have a relatively small voltage drop at 1 A/g. However, the TRGCE has an energy density of 62.5 Wh/kg, which is 2.7 folds higher than that of TRGC (23.1 Wh/kg). The capacitance of the TRGCE is 2.1 folds higher than that of the TRGC, suggesting that the increase of energy density is mainly attributed to the increased capacitance. On the other hand, the energy density of the TRG drops quickly with the current density as a result of its small capacitance and large initial voltage drop and that limited its energy density of 0.3 Wh/kg at 6 A/g. Further, at a high discharge current of 18 A/g, it is found that TRGCE (12.6 Wh/kg) still has a larger energy density than the TRGC (10.4 Wh/kg).

## 4. Conclusions

In summary, we investigate the influence of the interlayer distance on the performance of the TRG based EDLC cells. The intercalation of CTAB in TRGC sheets results in an interlayer distance of 0.41 nm, which is not sufficiently large for the EMI-TFSI ionic liquid. A facile approach is demonstrated to further enlarge the interlayer distance between TRGCE sheets by the ionic interaction between the intercalated CTAB and the EMI-TFSI. It is found that for the TRGCE, the vibration mode of the intercalated CTAB was shifted due to the ionic interaction with the additional EMI-TFSI. In addition, the X-ray data reveal that the interlayer distance of the TRGCE is enlarged to 2.51 nm. The results above suggest the formation of large ionic aggregates or micelles in between TRGCE sheets. Further, the TRGCE cell has the highest energy density of 62.5 Wh/kg, which is 2.7 folds higher than that of the TRGC cell (23.1 Wh/kg). The TRGCE cell (141 F/g) has a capacitance which is 2.1 folds higher than that of the TRGC cell (67 F/g), suggesting that the increase in energy density of the TRGCE cell is mainly attributed to the increased interlayer distance that facilitates the ion transport through pores and hence increases the accessible surface area for the ionic liquid electrolyte.

## References

[1] Wang G.P., Zhang L., Zhang J.J. (2012). A review of electrode materials for electrochemical supercapacitors. Chem. Soc. Rev..

[2] Zhang L.L., Zhao X.S. (2009). Carbon-based materials as supercapacitor electrodes. Chem. Soc. Rev..

[3] Zhu Y., Murali S., Cai W., Li X., Suk J.W., Potts J.R., Ruoff R.S. (2010). Graphene and Graphene Oxide: Synthesis, Properties, and Applications. Adv. Mater..

[4] Li X., Cai W., An J., Kim S., Nah J., Yang D., Piner R., Velamakanni A., Jung I., Tutuc E. (2009). Large-Area Synthesis of High-Quality and Uniform Graphene Films on Copper Foils. Science.

[5] Reina A., Jia X., Ho J., Nezich D., Son H., Bulovic V., Dresselhaus M.S., Kong J. (2008). Large Area, Few-Layer Graphene Films on Arbitrary Substrates by Chemical Vapor Deposition. Nano Lett..

[6] Karmakar S., Kulkarni N.V., Nawale A.B., Lalla N.P., Mishra R., Sathe V.G., Bhoraskar S.V., Das A.K. (2009). A novel approach towards selective bulk synthesis of few-layer graphenes in an electric arc. J. Phys. D Appl. Phys..

[7] Sutter P.W., Flege J.I., Sutter E.A. (2008). Epitaxial graphene on ruthenium. Nat. Mater..

[8] Marcano D.C., Kosynkin D.V., Berlin J.M., Sinitskii A., Sun Z., Slesarev A., Alemany L.B., Lu W., Tour J.M. (2010). Improved Synthesis of Graphene Oxide. ACS Nano.

[9] Stankovich S., Dikin D.A., Piner R.D., Kohlhaas K.A., Kleinhammes A., Jia Y., Wu Y., Nguyen S.T., Ruoff R.S. (2007). Synthesis of graphene-based nanosheets via chemical reduction of exfoliated graphite oxide. Carbon.

[10] Tan C., Cao X., Wu X.J., He Q., Yang J., Zhang X., Chen J., Zhao W., Han S., Nam G.H. (2017). Recent Advances in Ultrathin Two-Dimensional Nanomaterials. Chem. Rev..

[11] Mao L., Zhang K., Chan H.S.O., Wu J.S. (2012). Surfactant-stabilized graphene/polyaniline nanofiber composites for high performance supercapacitor electrode. J. Mater. Chem..

[12] Zhang K., Mao L., Zhang L.L., Chan H.S.O., Zhao X.S., Wu J.S. (2011). Surfactant-intercalated, chemically reduced graphene oxide for high performance supercapacitor electrodes. J. Mater. Chem..

[13] Meng W., Gall E., Ke F., Zeng Z., Kopchick B., Timsina R., Qiu X. (2015). Structure and Interaction of Graphene Oxide-Cetyltrimethylammonium Bromide Complexation. J. Phys. Chem. C.

[14] Largeot C., Portet C., Chmiola J., Taberna P.L., Gogotsi Y., Simon P. (2008). Relation between the ion size and pore size for an electric double-layer capacitor. J. Am. Chem. Soc..

[15] Fouda A.N., Assy M.K.A., el Enany G., Yousf N. (2015). Enhanced Capacitance of Thermally Reduced Hexagonal Graphene Oxide for High Performance Supercapacitor. Fuller. Nanotub. Carbon Nanostruct..

[16] Kim B.C., Cho W.J., Lee W.G., Kim S.J., Jalili R., Park S.Y., Wallace G.G., Yu K.H., Chang S.J. (2014). Capacitive behaviour of thermally reduced graphene oxide in a novel ionic liquid containing di-cationic charge. Synth. Met..

[17] Chen L.G., Lerum R.V., Aranda-Espinoza H., Bermudez H. (2010). Surfactant-Mediated Ion Exchange and Charge Reversal at Ionic Liquid Interfaces. J. Phys. Chem. B.

[18] Gong Y.N., Li D.L., Fu Q., Pan C.X. (2015). Influence of graphene microstructures on electrochemical performance for supercapacitors. Prog. Nat. Sci. Mater. Int..

[19] Ramimoghadam D., Hussein M.Z.B., Taufiq-Yap Y.H. (2012). The Effect of Sodium Dodecyl Sulfate (SDS) and Cetyltrimethylammonium Bromide (CTAB) on the Properties of ZnO Synthesized by Hydrothermal Method. Int. J. Mol. Sci..

[20] Sau T.K., Murphy C.J. (2005). Self-assembly patterns formed upon solvent evaporation of aqueous cetyltrimethylammonium bromide-coated gold nanoparticles of various shapes. Langmuir.

[21] Suteewong T., Sai H., Lee J., Bradbury M., Hyeon T., Gruner S.M., Wiesner U. (2010). Ordered mesoporous silica nanoparticles with and without embedded iron oxide nanoparticles: Structure evolution during synthesis. J. Mater. Chem..

[22] Taberna P.L., Simon P., Fauvarque J.F. (2003). Electrochemical characteristics and impedance spectroscopy studies of carbon-carbon supercapacitors. J. Electrochem. Soc..

[23] Lewandowski A., Olejniczak A., Galinski M., Stepniak I. (2010). Performance of carbon-carbon supercapacitors based on organic, aqueous and ionic liquid electrolytes. J. Power Sources.

[24] Kotz R., Carlen M. (2000). Principles and applications of electrochemical capacitors. Electrochim. Acta.

[25] Pandey G.P., Rastogi A.C. (2012). Solid-State Supercapacitors Based on Pulse Polymerized Poly(3,4-ethylenedioxythiophene) Electrodes and Ionic Liquid Gel Polymer Electrolyte. J. Electrochem. Soc..

[26] Liu R., Cho S.I., Lee S.B. (2008). Poly(3,4-ethylenedioxythiophene) nanotubes as electrode materials for a high-powered supercapacitor. Nanotechnology.

[27] Yoon S., Lee J.W., Hyeon T., Oh S.M. (2000). Electric double-layer capacitor performance of a new mesoporous carbon. J. Electrochem. Soc..

[28] Mo Y.F., Wan Y.F., Chau A., Huang F.C. (2014). Graphene/Ionic Liquid Composite Films and Ion Exchange. Sci. Rep..

[29] Du M., Yang T., Ma S.Y., Zhao C.Z., Jiao K. (2011). Ionic liquid-functionalized graphene as modifier for electrochemical and electrocatalytic improvement: Comparison of different carbon electrodes. Anal. Chim. Acta.

[30] Zhu Y., Murali S., Stoller M.D., Ganesh K.J., Cai W., Ferreira P.J., Pirkle A., Wallace R.M., Cychosz K.A., Thommes M. (2011). Carbon-Based Supercapacitors Produced by Activation of Graphene. Science.

[31] Tamilarasan P., Ramaprabhu S. (2013). Graphene based all-solid-state supercapacitors with ionic liquid incorporated polyacrylonitrile electrolyte. Energy.

[32] Yang H., Kannappan S., Pandian A.S., Jang J.H., Lee Y.S., Lu W. (2017). Graphene supercapacitor with both high power and energy density. Nanotechnology.

